# Transforaminal epidural steroid injection combined with radio frequency for the treatment of lumbar disc herniation: a 2-year follow-up

**DOI:** 10.1186/s12891-021-04209-5

**Published:** 2021-04-12

**Authors:** Wen-Bo Wei, Sha-Jie Dang, Ling Wei, Tian Liu, Jue Wang

**Affiliations:** 1 The Key Laboratory of Biomedical Information Engineering of Ministry of Education, The Key Laboratory of Neuro-informatics & Rehabilitation Engineering of Ministry of Civil Affairs, and Institute of Health and Rehabilitation Science, School of Life Science and Technology, Xi’an Jiaotong University, Xi’an, 710049 P. R. China; 2 National Engineering Research Center of Health Care and Medical Devices, Guangzhou, China; 3 Department of orthopedics, Shaanxi Provincial people’s Hospital, Xi’an, Shaanxi China; 4 Department of Anesthesia, Shaanxi Provincial Cancer Hospital, Xi’an, Shaanxi China; 5 Department of Pain, YangLing Demonstration Zone Hospital, Yangling, Shaanxi China

**Keywords:** Transforaminal epidural steroid injection, Radio frequency, Lumbar disc herniation

## Abstract

**Background:**

To assess the therapeutic efficacy of transforaminal epidural steroid injection (TFESI) combined with radio frequency (RF) for the treatment of lumbar disc herniation (LDH).

**Methods:**

A total of 230 patients participated in the study: TFESI (Group T, *n* = 110), TFESI combined with RF (Group TR, *n* = 120). Visual analogue scale (VAS), Oswestry disability index (ODI) and Global perceived effect (GPE) scale were measured pre-operation, 1, 3, 6, 12 and 24 months after the operation. Hospitalization time, treatment time, complications, and recurrence were compared between the two groups.

**Results:**

The VAS and ODI at each observation point of the post-operation were significantly decreased compared with the pre-operation in both groups (*P* < 0.05). There was no statistically difference of VAS and ODI between the two groups at 1 and 3 months of the post-operation (*P* > 0.05). However, The VAS and ODI scores in Group TR were significantly lower than that in Group T at 6, 12 and 24 months of the post-operation (*P* < 0.05). The GPE in group TR was high in the early days, while that at 1 and 3 months after treatment was significantly higher than that in group T (*P* < 0.05). The recurrence rate in Group TR was lower than that in Group T (*P* = 0.002). There was no significant difference in hospitalization time, complications, VAS and ODI score at the pre-operation between the two groups (*P* > 0.05).

**Conclusion:**

These findings suggest that TFESI combined with RF could effectively improve the pain and function, and had a long-term satisfactory effect for the treatment of LDH.

## Background

Lumbar radicular pain is caused by Lumbar disc herniation (LDH), which is a common orthopaedic disease characterized by low back pain and sciatica. The incidence rate is 10–20% [1] and has become a global health issue [2]. There are many treatment methods for LDH, including conservative treatment, interventional and surgery therapy. Clinically, patients with LDH who had no effect of conservative treatment undergo invasive treatment. Choosing an invasive therapy that has fewer traumas, maintains the integrity and stability of the spine, and reduces the incidence of postoperative complications has become the focus of LDH treatment.

Transforaminal epidural hormone injection (TFESI), as a minimally invasive interventional surgery, is widely used in the treatment of LDH [3]. It has the advantages of less trauma, fewer complications, and faster onset. It relieves symptoms by injecting corticosteroids and local anesthetics around the dural and nerve roots that cause radicular pain. Previous studies have shown that TFESI has a positive short-term effect in reducing lumbar back pain. However, the medium- and long-term treatment efficiency of TFESI is unsatisfactory [3, 4].

Radio frequency (RF) is one of the interventional therapies for LDH, which uses radio frequency alternating current to ablate the tissue around the needle electrode [5]. Radio frequency can be used as a means that targets certain anatomical structures of interest, usually nerves. Some results show that the medium- and long-term treatment efficiency is satisfactory [6, 7].

We hypothesized that TFESI combined with RF could lead to a significant reduction in pain related to LDH for a long-term. The present study aimed to retrospectively analyze the 2-year follow-up data after TFESI combined with RF for the treatment of LDH in YangLing Demonstration Zone Hospital, and evaluate the effectiveness and safety through clinical assessment tools and patient interviews.

## Methods

### Study design

We retrospectively reviewed the medical records of 230 patients, who were treated at the Department of Pain Management, YangLing Demonstration Zone Hospital between January 2014 and December 2016. This study was approved by the clinical research ethics committee of YangLing Demonstration Zone Hospital (No. 2016–021). This study followed the Good Clinical Practice guidelines and the guidelines of the Helsinki Declaration. The study included 110 cases that received TFESI (Group T) and 120 cases that received TFESI combined with RF (Group TR).

### Patients

**Fig. 1:**
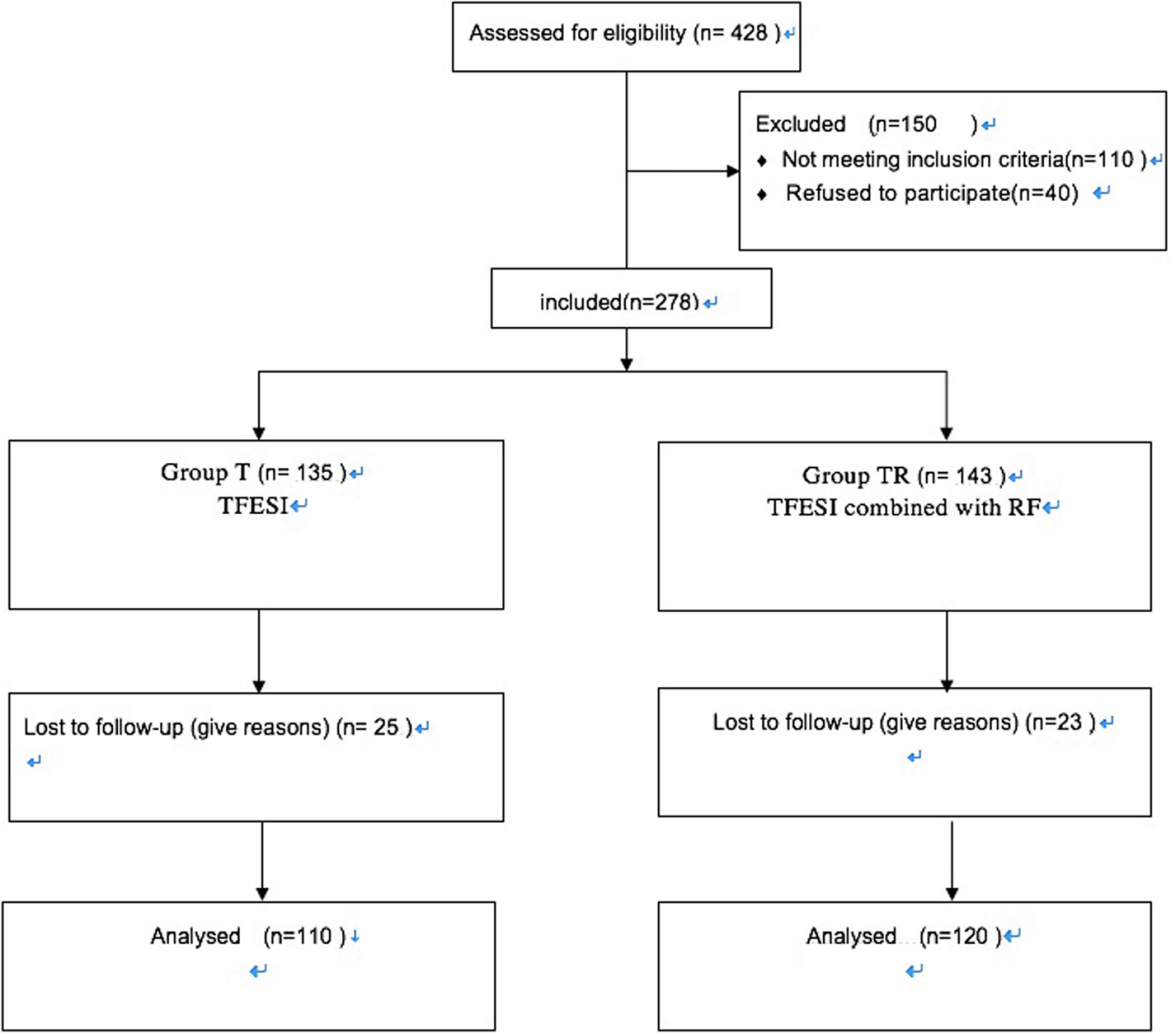
Schematic illustration of the study design. Note: all 230 patients were included in the treatment. Abbreviations: TFESI, transforaminal epidural steroid injection; RF, radio frequency

Patients (aged 21 ~ 70 years old, BMI 16 ~ 38 and ASAI~III) undergoing TFESI combined with RF or TFESI operation as LDH were screened in this study (Fig. 1). All patients presented who refused open surgery with lower back pain with sciatica and were ineffective after 3 months of adequately conservative treatment. The patient’s signs and symptoms are caused by herniated discs, which was confirmed by MRI and CT. All patients were recorded CTF-classification of LDH [8]. The following patients were excluded: multi-segmental disc herniation, sequestration type disc herniation, cauda equina syndrome, lumbar spinal stenosis, spinal metastatic disease, lumbar spondylolisthesis, psychosis, uncorrectable bleeding quality, patients who lost to follow-up, and previous lumbar surgery.

### Procedures

Guided by C-arm fluoroscopy, the patient is placed in the prone position with U-shaped pillows under the chest and both ilia so that the abdomen is suspended. The injection site was sterilized with antiseptic fluid and draped with surgical towels.

**Fig. 2:**
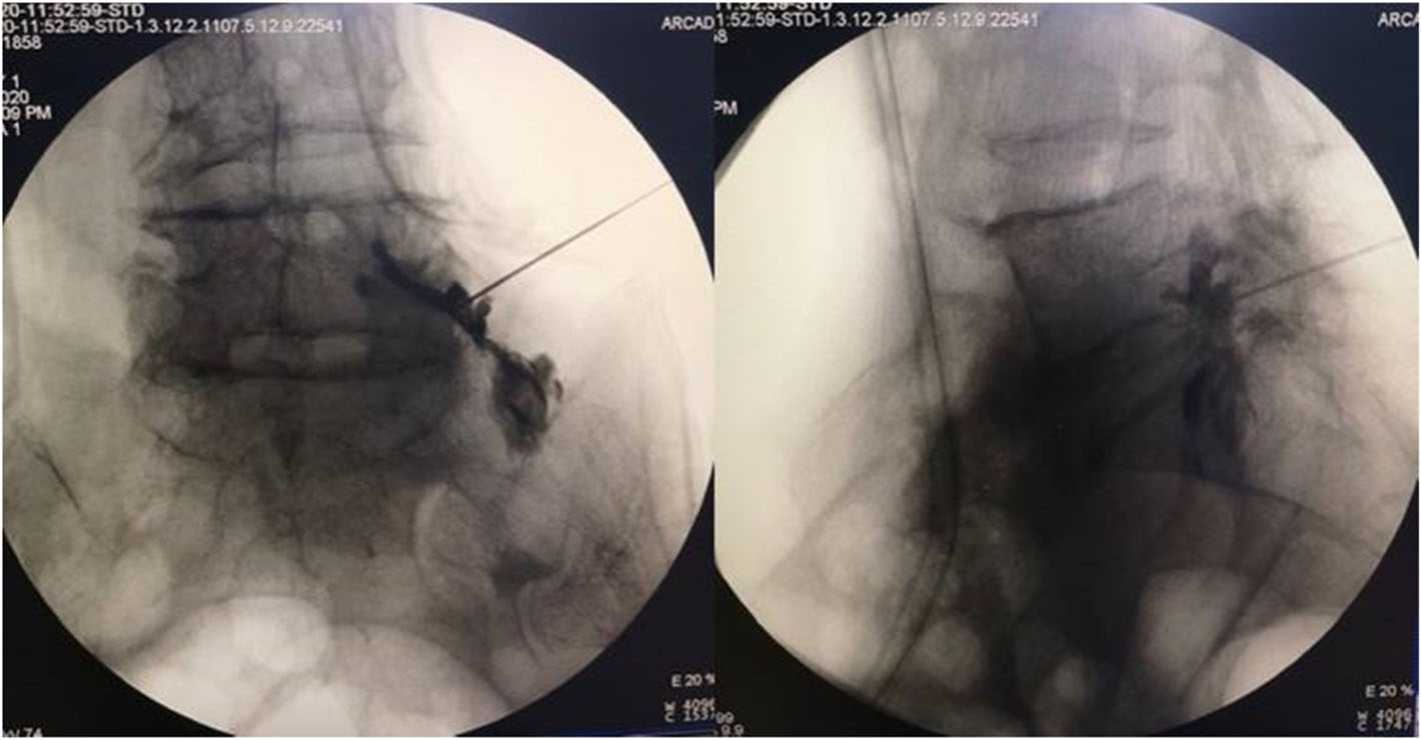
Anteroposterior and lateral C-arm images with needle placement and spread of dye along the nerve root

In the TFESI procedure, a local anesthetic (3 mL of 0.5% lidocaine) is injected into the skin and subcutaneous tissue at the injection site. A 0.35-in. 18 cm needle is advanced in the area below the pedicle. As the epidural space is approached, anteroposterior and lateral view will be taken to confirm the needle position. 0.5 mL contrast medium was used to check whether the needle was in the epidural space. 3 mL mixture of corticosteroids and anesthetics (80 mg methylprednisolone, 5 mL 2% lidocaine and 5 mL 1% ropivacaine) were injected (Fig. 2).

**Fig. 3:**
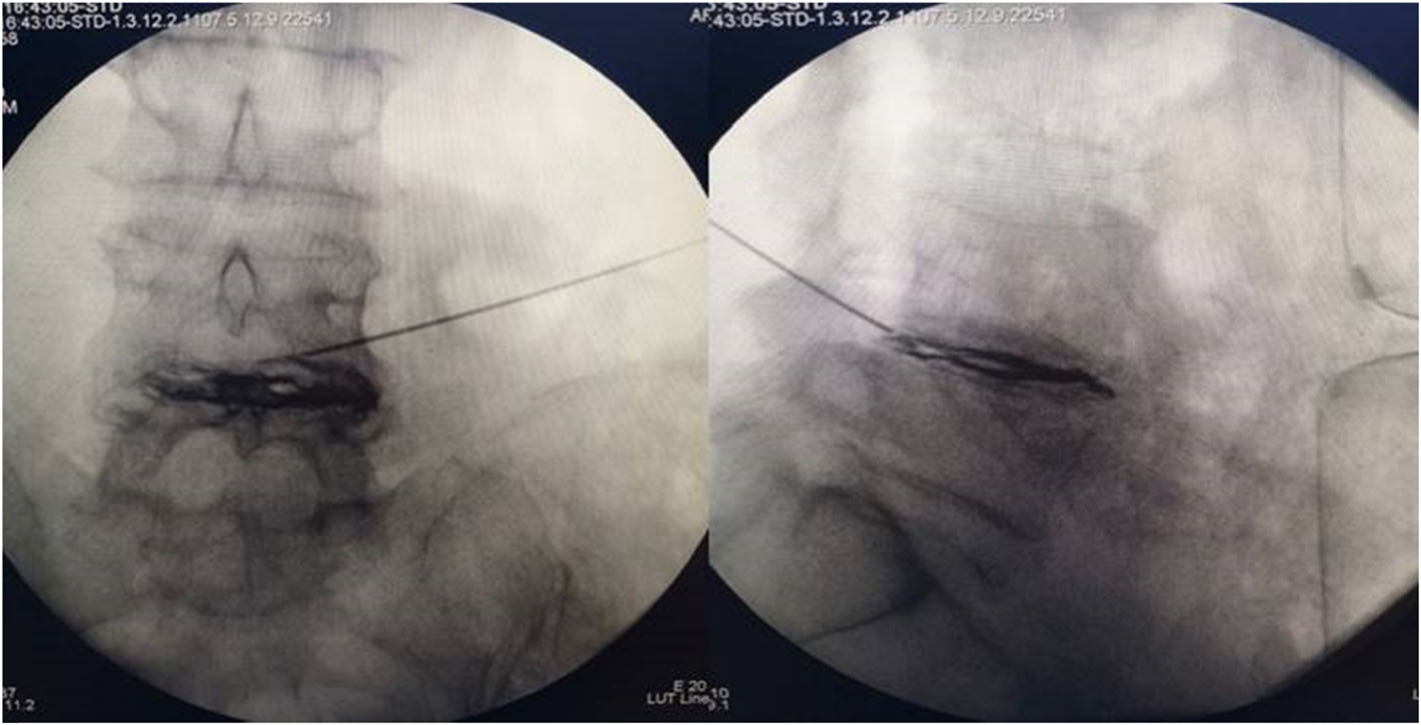
Anteroposterior and lateral C-arm images with needle placement and dye spreading along the disc

In the RF procedure, a puncture needle using a radio frequency probe is used to pierce the protrusion. According to the preoperative physical examination and imaging examination, the responsible target position of the intervertebral disc was determined. 6-14 cm was opened beside the posterior midline. The puncture was carried out through the safety triangle with an included angle of 20–40 at the coronal position to the herniated part of the intervertebral disc. After confirming the position of the guide needle with the anteroposterior and lateral view, we remove the probe, use or not 1 mL contrast medium to check whether the needle is in the disc, then advance the catheter rod through the guide needle to the center of the protruding portion. Radio frequency treatment was performed using a radio frequency temperature-controlled thermocouple (XJ-08; Xi’an Sterilization Equipment Manufacturing Co., Ltd.; Xi’an). The electrodes are inserted into a puncture needle, sensory and motor responses during RF neurotomy procedure might be used (albeit not obligatorily) to confirm the close proximity of targeted (and non-targeted) nerves to RF electrodes. Thermo coagulation was applied at 60 °C, 70 °C, and 80°Cfor 60s each, and 90 °C for 100 s. (Fig. 3).

Sex, age, BMI, operating time, and hospitalization time were evaluated and recorded. Follow-ups were conducted preoperatively, and at 1, 3, 6, 12 and 24 months postoperatively. During the individual interviews, we collected information from the patients regarding side effects, discomfort, and recurrence. Assessments were conducted using the visual analog scale (VAS) [9], the Oswestry Disability Index (ODI) [10], andthe Global perceived effect (GPE) [11]. The VAS rates pain severity as a score from 0 to 10, 0 indicates no pain and a score of 10 indicates the most severe pain. The ODI assesses low back pain-related disability: the higher the score, the more severe the disability. The Global perceived effect (GPE) scale is a 7-question scale that asks subjects to rate their condition after receiving treatment, where 1 = worst ever and 7 = best ever. Success was defined as a score ≥ 5. Recurrent disc herniation is defined as a painless interval of at least 6 months after surgery, with herniated disc material at the same level and presenting the same symptoms as before surgery [12].

### Outcomes

The primary outcomes were VAS and ODI pre-operation, 1, 3, 6, 12, and 24 months after the operation. The secondary outcomes included operation time, hospitalization time, complication and recurrence.

### Statistical analysis

The statistical analysis was performed with SPSS 24.0 for Windows (SPSS, Inc., IBM). Measured data were tested for normal distribution and the homogeneity of variance. Numeric variables were expressed as Mean ± SD and analyzed by Independent-Samples T-test. Repeated measures of ANOVA (with Bonferroni confidence interval adjustment) tests were conducted for VAS and ODI. Categorical data were expressed by N (%) and were analyzed with the χ2 test. The value of *p* < 0.05 was taken as a significant difference.

Sensitivity analysis was performed to account for the patients who didn’t receive open surgery during the 2-year study period. The recorded value was excluded, as for the patients who underwent open surgery.

## Results

### General information

There was no significant difference in the sex, age, BMI, classification of disc herniation and hospitalization time between the two groups (*P* > 0.05). Treatment time in Group TR (63.42 ± 9.79) was significantly longer than that in Group T (29.02 ± 7.49) (*P* < 0.001). The occurrence of recurrence rates in Group TR (34.17%) was lower than that in Group T (54.55%) (*P* = 0.002). During the 2-year follow-up, in Group T, 38 patients underwent open surgery; and in Group TR, 25 patients underwent open surgery. No severe complications, such as spinal injury and paraplegia, occurred in the patients in the three groups. In Group T, nerve root injury occurred in 2 patients. In Group TR, nerve root injury occurred in 4 patients, dural puncture occurred in 2 patients. The complications were reversible and resolved within 3 months. The complication rate during follow-up had no significant difference between the two groups (*P* > 0.05) (Table 1).

**Table 1:** Comparison of general data between Group T and Group TR

		Group T (*n* = 110)	Group TR (*n* = 120)	t/ (x^2^)	*P*
Male/female		47/63	53/67	(0.048)	0.826
Age (years)		64.70 ± 14.23	65.54 ± 16.06	−0.419	0.675
BMI (kg/m^2^)		24.48 ± 5.05	24.43 ± 5.20	0.077	0.939
Disc herniation classification	Protrusion	62	66	(0.043)	0.835
	Extrusion	48	54		
Hospitalization time (days)		5.39 ± 1.24	5.88 ± 2.43	−1.907	0.058
Operation time (min)		29.02 ± 7.49	63.42 ± 9.79	−30.092	< 0.001*
Complication		2 (1.82%)	6 (5%)	(0.913)	0.339
Recurrence		60 (54.55%)	41 (34.17%)	(9.677)	0.002*

*Notes*: Numeric data were expressed as Mean ± SD and analyzed by Independent-Samples T-test. Categorical data were expressed by the number of patients (%) and were analyzed with the χ2 test. Group T: TFESI group; Group TR: TFESI combined with RF group. *P < 0.05, Group T vs Group TR

*Abbreviations*: *BMI* body mass index, *VAS* visual analog scale, *ODI* Oswestry Disability Index, *TFESI* transforaminal epidural steroid injection, *RF* radio frequency

### Comparison of VAS

There was no significant difference in the postoperative low back pain and lower limb radicular pain (VAS score) at the pre-operation between the two groups. The VAS of the low back pain and lower limb radicular pain at each observation point of the post-operation were significantly decreased compared with the pre-operation in both groups (*P* < 0.05). There was no statistical difference of VAS between the two groups at 1 and 3 months of the post-operation (*P* > 0.05). However, The VAS scores in Group TR were significantly lower than that in Group T at 6, 12 and 24 months of the post-operation (*P* < 0.05).

### Comparison of ODI

There was no significant difference in ODI score at the pre-operation between the two groups. The ODI of the low back pain and sciatica at each observation point of the post-operation were significantly decreased compared with the pre-operation in both groups (*P* < 0.05). There was no statistical difference in ODI between the two groups at 1 and 3 months of the post-operation (*P* > 0.05). However, The ODI scores in Group TR were significantly lower than that in Group T at 6, 12 and 24 months of the post-operation (*P* < 0.05).

### Comparison of GPE

The percentage of patients with GPE ≥5 was shown in Table 2. The GPE in group A decreased with time; it was significantly lower at 6,12 and 24 months after treatment than that in group B (*P* < 0.05). The GPE in group B was higher in the early days, while that at 3 months after treatment was significantly higher than group A (*P* < 0.05) (Table 2).

**Table 2:** Comparison of GPE after treatment in the two groups (n, %)

Time		Group		*P*
		T (*n* = 110)	TR (*n* = 120)	
1 month		88 (80.0%)	98 (81.7%)	0.715
3 months		83 (75.5%)	99 (82.5%)	0.042
6 months		78 (70.9%)	91 (75.8%)	0.008
12 months		61 (55.5%)	82 (68.3%)	0.015
24 months	Total	49 (44.5%)	76 (63.3%)	0.030
	Without further surgery	21 (29%)*n* = 72	56 (58%) *n* = 95	0.013
	After open surgery	28 (80%)*n* = 38	20 (80%) *n* = 25	0.577

*Notes*: GPE results: percentages of patients with score ≥ 5. Data are presented as numbers (%) of patients. Group T: TFESI; Group TR: TFESI combined with RF. Group T compared to Group TR, *P < 0.05

*Abbreviations*: *GPE* global perceived effect, *TFESI* transforaminal epidural steroid injection, *RF* radio frequency

### Sensitivity analysis

Patients who received open surgery during the 2-year study period were excluded. There was no significant difference in ODI score and VAS score at the pre-operation between the two groups. The ODI of the low back pain and sciatica and the VAS of the low back pain and lower limb radicular pain at each observation point of the post-operation were significantly decreased compared with the pre-operation in both groups (*P* < 0.05). There was no statistical difference of ODI and VAS between the two groups at 1 and 3 months of the post-operation (*P* > 0.05). However, The ODI scores and VAS scores in Group TR were significantly lower than those in Group T at 6, 12 and 24 months of the post-operation (P < 0.05). The results were similar to those without exclusion. (Tables 3 and 4).

**Table 3:** Comparison of VAS between Group T and Group TR without further surgery at different time

Group		Pre-operation	Post-operation				
			1 month	3 months	6 months	12 months	24 months
Group T (*n* = 72)	low back pain	7.04 ± 1.02	2.10 ± 0.96^a^	2.38 ± 0.97^a^	2.66 ± 1.07^a^	3.27 ± 1.02^a^	3.33 ± 0.93^a^
	sciatica	7.59 ± 1.01	2.10 ± 0.99^a^	2.37 ± 0.96^a^	2.67 ± 1.07^a^	3.21 ± 0.96^a^	3.14 ± 0.89^a^
Group TR (*n* = 95)	low back pain	7.12 ± 0.98	2.16 ± 0.87^a^	2.01 ± 0.90^a^	1.96 ± 0.78^ab^	2.20 ± 0.91^ab^	2.13 ± 0.72^ab^
	sciatica	7.52 ± 1.04	2.15 ± 0.87^a^	2.10 ± 0.91^a^	1.98 ± 0.79^ab^	2.17 ± 0.91^ab^	2.38 ± 0.71^ab^
Time F, *P*	low back pain	661.957,< 0.001					
	sciatica	666.713,< 0.001					
Group F, *P*	low back pain	30.212, < 0.001					
	sciatica	30.356, < 0.001					
Time * Group F, *P*	low back pain	18.496, =0.001					
	sciatica	14.635, < 0.001					

*Notes*: Patients who received open surgery during the 2-year study period were deleted. Data are presented as mean ± SD. The groups were compared by repeated measures analysis of variance (ANOVA). Bonferroni correction was used to correct multiple comparisons. Group T: TFESI group; Group TF: TFESI combined with RF group; vs pre-operation in the same group, ^a^*P* < 0.05; vs Group T in the same time, ^b^*P* < 0.05

*Abbreviations*: *VAS* visual analog scale, *TFESI* transforaminal epidural steroid injection, *RF* radio frequency

**Table 4:** Comparison of ODI between Group T and Group TR without further surgery at different time

Group	Pre-operation	Post-operation				
		1 month	3 months	6 months	12 months	24 months
Group T (n = 72)	69.45 ± 6.71	14.90 ± 3.90^a^	16.12 ± 4.39^a^	26.12 ± 6.05^a^	26.11 ± 8.92^a^	27.77 ± 8.46^a^
Group TR (n = 95)	70.76 ± 6.68	15.27 ± 3.86^a^	15.54 ± 5.37^a^	15.68 ± 4.61^ab^	19.19 ± 6.34^ab^	20.91 ± 7.20^ab^
Time F, *P*	1825.276, < 0.001					
Group F, *P*	94.292, < 0.001					
Time * Group F, *P*	33.229, < 0.001					

*Notes*: Patients who received open surgery during the 2-year study period were deleted. Data are presented as mean ± SD. The groups were compared by repeated measures analysis of variance (ANOVA). Bonferroni correction was used to correct multiple comparisons. Group T: TFESI group; Group TF: TFESI combined with RF group; vs pre-operation in the same group, ^a^*P* < 0.05; vs Group T in the same time, ^b^*P* < 0.05

*Abbreviations*: *ODI* Oswestry Disability Index, *TFESI* transforaminal epidural steroid injection, *RF* radio frequency

## Discussion

LDH is one of the most common causes of low back pain and sciatica, it affects the daily life of patients. Therefore, there is an urgent need to alleviate the pain and improve the quality of life of these patients.

The mechanism of pain caused by LDH is multifaceted, may be caused by mechanical and/or inflammatory factors. Disc herniation can cause direct compression of nerve roots or dorsal root ganglia, as well as indirect compression of perineural blood vessels. Once the epidural tissue around the nerve root and the nerve root itself get inflamed and produce a series of inflammatory mediators, all of these mediators activate the afferent nerves, and make the nerves very sensitive to pressure and cause pain [13–16]. Currently, most LDH are treated by reducing stress and/or reducing the release of inflammatory factors. With the continuous development of spinal surgery technology, interventional therapy is increasingly performed due to its many advantages over open surgery, including minimal tissue trauma, fewer surgical complications, and earlier postoperative recovery.

TFESI is usually performed in patients with LDH. The goal is to deliver the drug directly to the damaged spinal nerve root. The most common drug is to use a mixture of local anesthetics and corticosteroids. Corticosteroids can inhibit the production and release of proinflammatory materials. Local anesthetics can inhibit the generation of action potentials, nerve impulses in response to noxious stimuli, and the transmission of pain stimuli to the brain. Several previous studies have demonstrated excellent short-term outcomes of TFESI in patients with LDH. It may result in improvement in lumbosacral radicular pain between two and six weeks, which may relate to the duration of the therapeutic effect of corticosteroid [17, 18]. Previous studies have reported that the short-term success rate is about 34–78% [19, 20]. However, many studies have shown controversial results about the long-term effects of the procedure [21, 22]. Pinto review analysis showed that epidural steroid injections have short-term effects on relieving low back pain and disability compared with placebo in patients with LDH but no effect in the long-term [23]. The present study showed that all patients had satisfactory clinical results six months after treatment in the two groups. In the TFESI group, we found some remarkable effects in the short term, but the long-term effect is not obvious, and the associated recurrence rate is higher than group B in this study. We analyze the reason that may be related to the local nerve compression has not been relieved, and local inflammatory factors regroup.

RF has been applied for the herniated disc of lumbar or cervical [24]. Through an electrode, an alternating current (frequency, 250–500 kHz) is produced by a radio frequency generator, causing ionic movements in the tissue directly surrounding the active tip. Under the condition of high temperature, RF ablation disrupts the molecular chains in nucleus pulposus tissue, leading to collagen shrinkage, nucleus pulposus degeneration, coagulation, atrophy, and reduction of disc total volume. Therefore, the intradiscal pressure was decreased and the stimulation of the nerve root was reduced. At the same time, RF can increase the local temperature in a short time. The thermal effects can improve local blood circulation, easing the inflammatory reaction of the rupture of the intervertebral disc near the nerve roots and within the spinal canal [25]. Many studies have shown that the long-term results were satisfactory. Nie provides a retrospective evaluation follow up five years, which found that RF can reduce pain in patients with lumbar disc herniation and improve quality of life in a long-term [7]. Our results indicate that in group TR, the VAS and ODI scores improved significantly over a longer period. TFESI combined with RF showed fast onset and long maintenance time. We considered the reason as follows: local anesthetics and cortisol drugs can alleviate pain in the short term; RF decompresses nerve roots and improves the internal environment around nerve roots, thereby achieving a long-term Analgesic effect.

Our research has several limitations. Firstly, this was a retrospective study. Therefore, there may be inherent bias associated with patient selection and missing patient information. Secondly, the study was performed in only one hospital with limited patients enrolled, larger scale clinical trial with multiple centers is needed in the future.

## Conclusion

The findings indicate that TFESI combined with RF for the treatment of LDH can effectively and rapidly relieve pain symptoms, improve quality of life, and have long-term satisfactory results but a 20% ratio of open surgery during follow-up must be anticipated.

## Data Availability

The authors will allow the sharing of participant data. The data will be available to anyone who wishes to access them for any purpose. The data will be accessible from immediately the following publication to 6 months after publication, and contact should be made via the first author by email.

## Abbreviations

TFESI: Transforaminal epidural steroid injection
RF: Radio frequency
LDH: Lumbar disc herniation
ASA: American Society of Anesthesiologists
VAS: Visual analog scale
GPE: Global perceived effect
ODI: Oswestry Disability Index
